# Unconserved C terminal of human cytomegalovirus tegument protein pUL76 elicits nuclear aggresome formation and induces DNA damage in transfected cells

**DOI:** 10.1186/s12929-015-0205-4

**Published:** 2015-10-22

**Authors:** Wenchang Zhang, Yao Yao, Jingxian Chen, Mingli Wang

**Affiliations:** Department of Microbiology, Anhui Medical University, 81st Meishan Road, Hefei, 230032 Anhui China; Department of Pathology & Cell Biology, Columbia University, New York, 10032 USA

**Keywords:** Human cytomegalovirus (HCMV), Tegument protein, UL76, Unconserved C terminal, Chromosome damage, Protein aggresome formation

## Abstract

**Background:**

The HCMV *UL76* gene is a member of *UL24* family in herpes virus and encodes a highly conserved herpes virus protein. Inherited from common ancestor, members of Herpes___UL24 family encode proteins with a conserved N terminal and varied in C terminal region. To define which region (conserved N terminal or unconserved C terminal) of UL76 was responsible for its ability to induce DNA damage and aggresome formation, the wild-type *UL76* gene and two deletion mutants were transfected to cells and analyzed by immunofluorescent staining, Western blotting and comet assay.

**Results:**

We report that the EGFP-fusion proteins present as globular aggresomes and colocalize with γ-H2AX in cells transfected with either pEGFP-UL76 or pEGFP-UL76C. The relative expression level of γ-H2AX and percentage of cells with comet tails were elevated in pEGFP-UL76 or pEGFP-UL76C transfection groups compared to the control.

**Conclusion:**

Our findings suggest that the unconserved C terminal (not the conserved N terminal) of pUL76 was sufficient to induce DNA damage and aggresome formation in transfected cells.

## Background

Human cytomegalovirus belongs to *Betaherpesvirus*. It has the largest size of genome among human herpesviruses, with an about 235 kb double strands DNA genome [1]. HCMV is a pathogen causing significant morbidity and mortality in populations with immature or compromised immune systems [2]. It is also the leading viral cause of birth defects, congenital HCMV infection causes deafness and mental retardation [3]. Like other herpesviruses it established lifelong latency after primary infection partly because the virus has evolved a series of strategies to counteract the suppression effect of host immune system. HCMV is able to modulate both the innate and adaptive immunity of the host [4, 5]. Studies of many viruses revealed their ability to nonspecifically induce cytogenetic damage to the chromosome of host cells [6]. DNA damage responses and repair pathways in host cells are activated after infection. DNA damage, especially double strands DNA breaks have a profound effect on stability of the genome.

Aberrant or misfolded proteins often cause adverse cell stresses and therefore need to be recognized immediately and removed efficiently by the cells. Accumulation or persistence of aberrant proteins within the cells often has deleterious consequences. For example, aberrant proteins may lose regulation, form inactive complexes that compete with functional complexes, assemble into aggregates that eliminate protein function or cause toxicity, or introduce harmful activities if mislocalized [7]. The accumulation of misfolded protein in aggregates has been described in some neurodegenerative diseases, such as Parkinson’s disease, Alzheimer’s disease and Creutzfeldt-Jakob disease [8].

HCMV infection induces site-specific chromosome damage at 1q23.3 and 1q42 in fibroblast. The DNA breaks in 1q23.3 between loci DFNA7 and DFNA49, were involved in dominantly inherited, sensorineural hearing impairment (SNHI) [9]. These observations suggested that cells infected with HCMV might provide a reservoir for genetic damage and, in a clinical perspective, a scenario could be envisioned whereby hearing impairment could result from early DNA damage of dividing fetal cells rather than viral replication and cell lysis. Interestingly, virus inactivated with UV was still capable of inducing chromosome damage even not site-specific. Viral entry into the cell was basically required. This suggested that components of the virus particles may be responsible for HCMV induced chromosome damage [6, 10]. More recently, studies from another group have demonstrated that UL76 of HCMV elicits aggresome formation and induces DNA damage in transfected cells [11, 12]. Although HCMV induced cellular DNA damage in the pathogenesis of HCMV infection is poorly understood, this was really apocalyptic.

The existing Herpes viruses are classified into three subfamilies, namely *Alphaherpesvirinae*, *Betaherpesvirinae* and *Gammaherpesvirinae*. Having millions years evolution with their host, herpes virus in different subfamilies have developed some distinct characteristics. The size of genome, cell tropism and replication cycle have varied from each other. However some genes are still conserved among subfamilies. These genes are likely to play essential roles in the biology of the virus [13]. Among which the *UL24* gene of human herpesvirus 1(HHV-1) defines a Herpes___UL24 family. Protein encoded by *UL76* of HCMV, a member of the conserved *UL24* gene family, has a conserved N terminal (1-190aa) and a varied C terminal region (187-325aa). Based on multiple protein sequence alignments of the Herpes___UL24 family, five conserved amino acid blocks were found in their N terminals. The amino acids of the blocks are as follows: block I, 19 to 35; block II, 67 to 82; block III, 97 to 106; block IV, 123 to 135; and block V, 151 to 162 [12]. One common role of Herpes___UL24 family members played during virus life cycle were defined by Nascimento [14]. They found that all representative members of Herpes___UL24 family induce cell cycle arrest by inactivation of the mitotic cyclinB/cdc2 complex. Other common roles were also suggested by sequence analysis. Using Meta-BASIC, Knizewski’s group identified that members of the *UL24* gene family encode a putative PD-(D/E)XK endonuclease domain [15]. For the purpose of defining new common roles of *UL24* family, some researchers set about to test the hypothesis that the highly conserved N terminal may function as the complete protein. This hypothesis was proved to be true by the research on the ability of UL24 for dispersal of the nucleolar protein nucleolin [16]. They also demonstrated that the conserved residues in the UL24 protein was crucial for its ability [17].

Inspired by their findings, we try to define which region could be decisive for UL76 to elicit aggresome formation and to induce DNA damage. Two truncate fragments, resembled the conserved N terminal (1-190aa) and unconserved C terminal (187-325aa) of UL76 respectively, were expressed in-frame with EGFP. We found that the unconserved C terminal, not the conserved N terminal, was sufficient in eliciting aggresome formation and inducing DNA damage.

## Methods

### Cell cultures

Human embryonic kidney (HEK293) cells were provided by professor Yan Liu, Anhui Medical University (Anhui province, China). Human embryonic lung fibroblasts (HELF) were purchased from the American Type Culture Collection (Manassas, VA). Cells were cultured in Dulbecco’s modified Eagle medium (DMEM) supplemented with 10 % heat-inactivated fetal bovine serum.

### Plasmid construction

The UL76 and its deletion constructs containing the N- and C-terminal regions were all derived by PCR amplification (the primers were listed in Table 1). The amplified DNA fragments, encoding full length of UL76, amino acids 1 to 190 and 187 to 325, were designed to contain the restriction endonuclease sites *Hind* III at the 5′ end and *BamH*I at the 3′ end. The PCR fragments were ligated to the linearized T vector pCR^@^ 2.1 (Invitrogen Carlsbad, CA, USA) and then transformed to bacterial DH5α. The resulting recombinant plasmids were designated T-UL76, T-UL76N, T-UL76C respectively. Both the vector pEGFP-N1 (Clontech) and the recombinant plasmids were doubly digested with *Hind* III and *BamH*I and then re-ligated. The eventual eukaryotic expression plasmids were named pEGFP-UL76, pEGFP-UL76N, pEGFP-UL76C respectively. Double digestion and sequencing were performed to verify the accuracy of recombinant plasmids construction.

**Table 1:** PCR primers used to amplify wild-type and deletion mutants of UL76. The size of products were list on the right. Restriction endonuclease sites are underlined in the primer sequences

	Primers	Product size/bp
UL76(1-325aa)	F: 5′-AAGCTTATGCCGTCCGGGCGT-3′	975
	R:5′-GGATCCGCTAAAGACCGTGTGGGACGGCG-3′	
UL76N(1-190aa)	F:5′- AAGCTTATGCCGTCCGGGCGT-3′	570
	R:5′- GGATCCGCCCGTCCCAGATAGTC-3′	
UL76C(187-325aa)	F:5′-AAGCTTATGGACTATCTGGGACGGCG-3′	420
	R:5′- GGATCCGCTAAAGACCGTGTGGGACGGCG-3′	

### DNA transfection and live illumination experiments

To analyze transient gene expression via live illumination, four different plasmids, pEGFP-N1, pEGFP-UL76, pEGFP-UL76N and pEGFP-UL76C, were transiently transfected to HEK293 cells respectively using Lipofectamine 2000 reagent (Invitrogen Carlsbad, CA, USA) according to the manufactures instruction. Living cell images were taken 48 h post transfection with Olympus DP73 fluorescence microscope.

### Flow cytometry

One day before transfection, HEK293 cells were seeded in 6-well plate at a concentration of 5 × 10^5^ /ml. Two μg of each plasmid DNA were transfected to HEK293 cells. At 24 h post transfection, cells were trypsinized, resuspended in PBS and adjusted to a concentration of 5 × 10^5^ cells/ml. To determine the transfection efficiency of each group, the EGFP positive cells were counted by flow cytometry (BD Bioscience, San Jose, CA, USA). In each test at least 1 × 10^5^ cells were counted.

### Western blot assay

To examine the production of EGFP-fusion proteins by western blotting, transfected cells were lysed in RIPA buffer (Beyotime, Shanghai, China) containing 1 mmol/L PMSF and protease inhibitor cocktail (Sigma-Aldrich) at an indicate concentration according to the manufacture instruction. Soluble cell lysate was collected and total protein concentration was determined by a Bradford protein assay kit (Bio-Rad, Hercules, CA, USA). Twenty microgram of total protein were separated by SDS-10 % PAGE and transferred to PVDF membrane. Membranes were blocked in TBST containing 3 % skim milk for one hour and then probed with the anti-GFP antibody (Beyotime, Shanghai, China) at 1:600 dilution overnight. After washed with TBST the membranes was incubated in TBST containing 3 % skim milk and 1:5000 diluted horseradish peroxidase-conjugated goat anti-rabbit IgG.

Western blotting to detect γ-H2AX was carried out by following the method described by Voon-Kwan Siew [11]. Anti-Phospho-Histone H2AX (Ser139) is a rabbit mAb (Cell Signaling Technology, Danvers, Massachusetts, USA) and the secondary antibody was the same as that for EGFP fusion proteins.

### Indirect immunofluorescent analyses

5 × 10^5^/ml HELF cells were seeded in 6-well culture plate in which coverslips were placed at the bottom 1 day before transfection. Plasmid DNA were transfected to HELF with Lipofectamine 2000 (Invitrogen, Carlsbad, CA, USA). Total amount of DNA for each transfection was maintained at a constant of 2 μg per well. At 24 h post transfection, cells were washed twice with PBS, fixed for 10 min with 4 % paraformaldehyde, treated with 0.1 % NP-40 in PBS for 30 min, and then incubated with γ-H2AX mAb (1:400), followed by donkey anti-rabbit IgG conjugated with Alexa Flour® 555 (Beyotime, Shanghai, China). Cells were co-stained with DAPI at a 1 ug/ml. Confocal images were acquired with a laser scanning confocal microscope (Leica DMI6000B).

### Comet assay

Comet assay was used to assess induction of DNA breaks by different recombinant plasmids in *vitro*. One day before transfection, 1 × 10^5^ cells /ml of HEK 293 cells were plated in 6-well culture plate. At 24 h post transfection, cells were harvested and combined to a concentration of 3 × 10^5^ cells/ml. Thirty μL of cell suspension were added to 70 μL of LMP (low melting point) agarose, and were then layered onto the slides pre-coated with NMP (normal melting point) agarose. Following lysis in alkaline lysis solution (2.5 M NaCl, 100 mM EDTA, 10 mM Tris, pH 10.0) for 1 h in 4 °C, slides were placed in gel box and treated with electrophoresis buffer (0.3 M NaOH/1 mM EDTA) for 20 min in dark to unwind, denature the DNA and hydrolyze the sites of damage. Cells were then subjected to alkaline electrophoresis at 1.0 volt cm^−1^ at 4 °C for one hour. The slides were treated with drops of neutralization buffer, and sit for at least 5 min. The neutralization was repeated two more times. After rinsing carefully with distilled water, the slides were dried and stained with DAPI.

### Statistical analyses

Statistical significance was assessed using the paired t test for independent samples. At least three independent experiments were evaluated for each transfection group and the mean ± standard deviation was given.

## Result

### Cloning and expression of full length and two truncated fragments of UL76

**Fig. 1:**
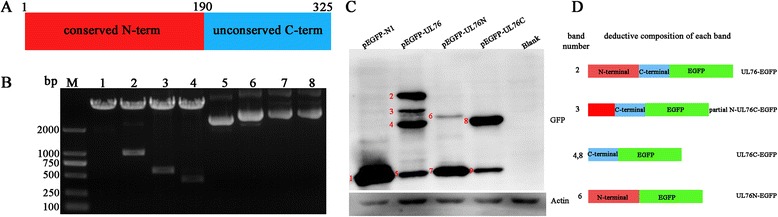
Cloning and expression of full-length and two deletion mutations of UL76. **a** Schematic depicting of composition of UL76. Amino acids 1–190 represent the conserved N terminal and amino acids 187–325 stand for the varied C terminal. **b** Double digestion to verify the accuracy of recombinant plasmids construction. Lane 1–4: Double digestion of pEGFP-N1, pEGFP-UL76, pEGFP-UL76N, pEGFP-UL76C respectively. Lane 5–8: Represent for pEGFP-N1, pEGFP-UL76, pEGFP-UL76N, pEGFP-UL76C respectively. **c** Western blot to detect different EGFP-fusion proteins. The predict MW of each fusion protein are: EGFP (27KD), EGFP-UL76 (63KD), EGFP-UL76N (49KD), EGFP-UL76C (41KD) respectively. Degradation of EGFP-tag from the fusion proteins were found in three transfection groups. Four bands have been detected in the pEGFP-UL76 group. This result indicated that there are three cleavage sites in the EGFP-UL76 protein. All bands presented on the membrane are numbered in order (from 1–9). **d** Deduced compositions of bands shown in **c**. Band 2 represents the full-length of UL76 and EGFP fusion protein. Band 3 indicates the cleavage happened in the early N-term of UL76, while band 4 stands for peptide cleaved between N-term and C-term of UL76. Since bands with predicted MW have been presented in each transfection group, the three recombinant plasmids were recognized as being expressed correctly

Primers were designed to amplify the full length and two truncated fragments of UL76 (Table 1). The amino acids 1–190 represent the conserved N terminal region of UL76, and amino acids 187–325 stand for the unconserved C terminal. PCR-produced DNA fragments were ligated to T Vector, and the resulting plasmids were named as T-UL76, T-UL76N, T-UL76C respectively. The inserts in the 3 recombinant plasmids were released from T vector, and subcloned to pEGFP-N1. Double digestion and DNA sequencing were performed to verify the sequence identity and proper orientation of the inserts in the pEGFP-N1 vectors. Frame shift mutations and nonsense mutations were excluded. Molecular weight of EGFP encode by pEGFP-N1 vector was 27 KD,the predict molecular weight of each EGFP fusion protein were:63 KD for UL76-EGFP, 49 KD for UL76N-EGFP and 41 KD for UL76C-EGFP. These recombinant plasmids and the empty vector were transfected to HEK293 cells respectively. At 48 h post transfection, cells were lysed and EGFP-tag antibody was used to detect fusion proteins encoded by the recombinant plasmids (Fig. 1c). EGFP-fusion proteins have been detected in each transfection group, but degradation of these proteins was also observed. The degradation was significant in pEGFP-UL76 and pEGFP-UL76N transfected groups. Degradation of these fusion proteins affected the measurement of their expression by WB. But products with the predict molecular weight (MW) of each group were all detected. This may partly confirm that fusion proteins were expressed correctly in our study. We reviewed the literatures regarding the using of EGFP/GFP for tagging and found that the tagging strategies strongly affect the fate of overexpressed protein. In a particular comparative assay [18], Bing Han et al. reported that the addition of fluorescent protein tags enhanced the degradation of caveolin-1, which is the primary scaffolding protein of caveolae.

### The efficiency of transfection was calculated by flow cytometry

**Fig. 2:**
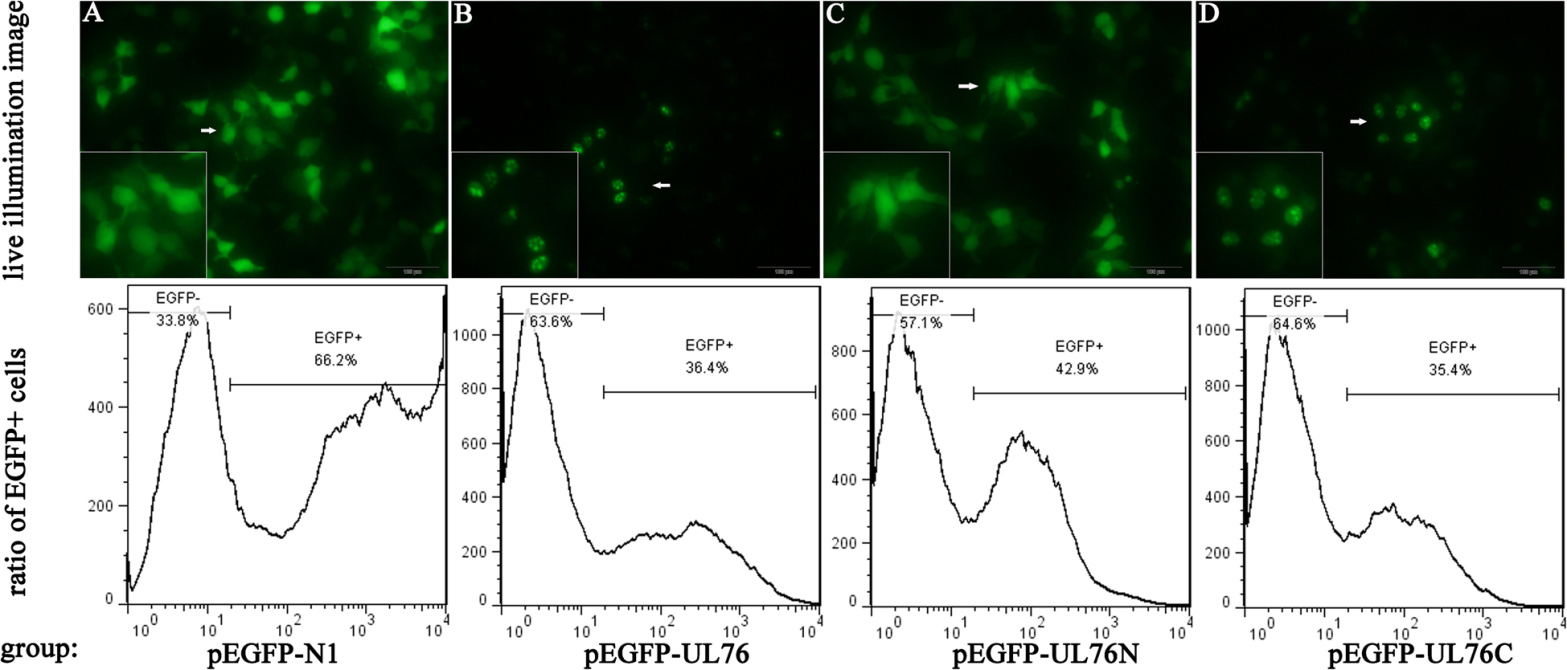
Assessment of the aggresome determinant region within UL76 (top panel) and transfection efficiency of each group (bottom panel). HEK293T cells were transiently expressed control EGFP and the fluorescent fusion proteins EGFP-UL76, EGFP-UL76N(1–190), and EGFP-UL76C(187–325). Fluorescent signals were accumulated into globular aggresome in cells transfected with pEGFP-UL76 (**b**) or pEGFP-UL76C (**d**). But the fluorescence intensities were diffusely distributed throughout most of the cells in pEGFP-N1 (**a**) and pEGFP-UL76N (**c**) groups. This indicates the unconserved C terminal of UL76 was the determinant region of its ability to elicit aggresome formation. The degradation of EGFP fusion proteins hampered our aim to measure the real expression level of UL76 and its deletion mutants by western blot. The ratio of EGFP positive cells in each transfection group may reflect the real expression level. Results of flow cytometry show that the EGFP+ ratio of each transfection group are: 66.2 % for pEGFP-N1, 36.4 % for pEGFP-UL76, 42.9 % for pEGFP-UL76N and 35.4 % for pEGFP-UL76C

Since the degradation of EGFP fusion proteins masked the expression level of UL76 and its truncated fragments, measurement of EGFP positive cells in each transfection group may reveal the efficiency of transfection. Our results showed that the pEGFP-N1 transfection group has the highest efficiency of 66.2 %, while the pEGFP-UL76N group has the efficiency of 42.9 %. The efficiency of pEGFP-UL76 and pEGFP-UL76C group were nearly equal, 36.4 % and 35.4 % respectively (Fig. 2).

### The unconserved C terminal of UL76 is the decisive region for elicitation of aggresome formation

To monitor the live image of fusion proteins, cells were transfected with pEGFP-UL76, pEGFP-UL76N and pEGFP-UL76C, and pEGFP-N1 was used as a negative control. After 48 h of transfection, images were acquired using a fluorescence microscope with emission between 505 nm and 540 nm for enhanced green fluorescent protein (EGFP). Interestingly, it was found that the unconserved C terminal of UL76 was the decisive region for its ability to elicit aggresome formation which opposite of previous observation [12] (Fig. 2). It was shown that both the full-length and unconserved C terminal of UL76 elicited aggresome formation whereas conserved N terminal and the pEGFP-N1 did not. Green fluorescence exhibited as globular aggresome in cells transfected with pEGFP-UL76 or pEGFP-UL76C. In contrast, cells transfected with pEGFP-N1 or pEGFP-UL76N showed diffuse fluorescence throughout most of the cells.

### The unconserved C terminal of UL76 is the decisive region of its ability to induce DNA damage in *vitro*

#### Indirect immunoflourescent assay

**Fig. 3:**
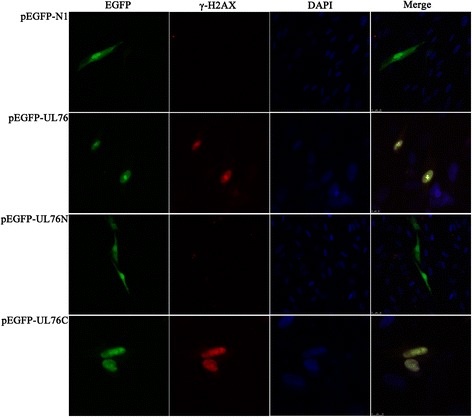
UL76 and its unconserved C terminal induced γ-H2AX foci. HELF cells were transfected with UL76, two deletion mutants or pEGFP-N1 (control vector). Colocalization of γ-H2AX (red) and EGFP fusion proteins (green) were found in nucleus of cells transfected with UL76 (row 2) and unconserved C terminal of UL76 (row 4), but not in cells transfected with pEGFP-N1 (row 1) or pEGFP-UL76N (row 3). Nuclei were counterstained with DAPI (blue)

H2AX is a family member of histone H2A, and comprises of 2–10 % of total histone H2A [19]. Phosphorylation of H2AX, also called γ-H2AX, is an initial cellular response to DNA double strand breaks. H2AX was phosphorylated by PI3K family members including ataxia-telangiectasia mutated gene (ATM), Rad3-related protein (ATR) and DNA-PK at Serine 139 which was conserved from *giardia intestinalis* to mammalian. The recruitment of γ- H2AX to the break point develops into visible foci in the nucleus. Different plasmids were transfected to HELF cells, indirect immunoflourescent assay was performed to detect colocalization of EGFP fusion proteins and γ-H2AX. Here we demonstrated that the γ-H2AX was colocalized with EGFP-UL76 or EGFP-UL76C, but it was rare to be seen in cells transfected with pEGFP-N1 or pEGFP-UL76N (Fig. 3).

#### Western bolting

**Fig. 4:**
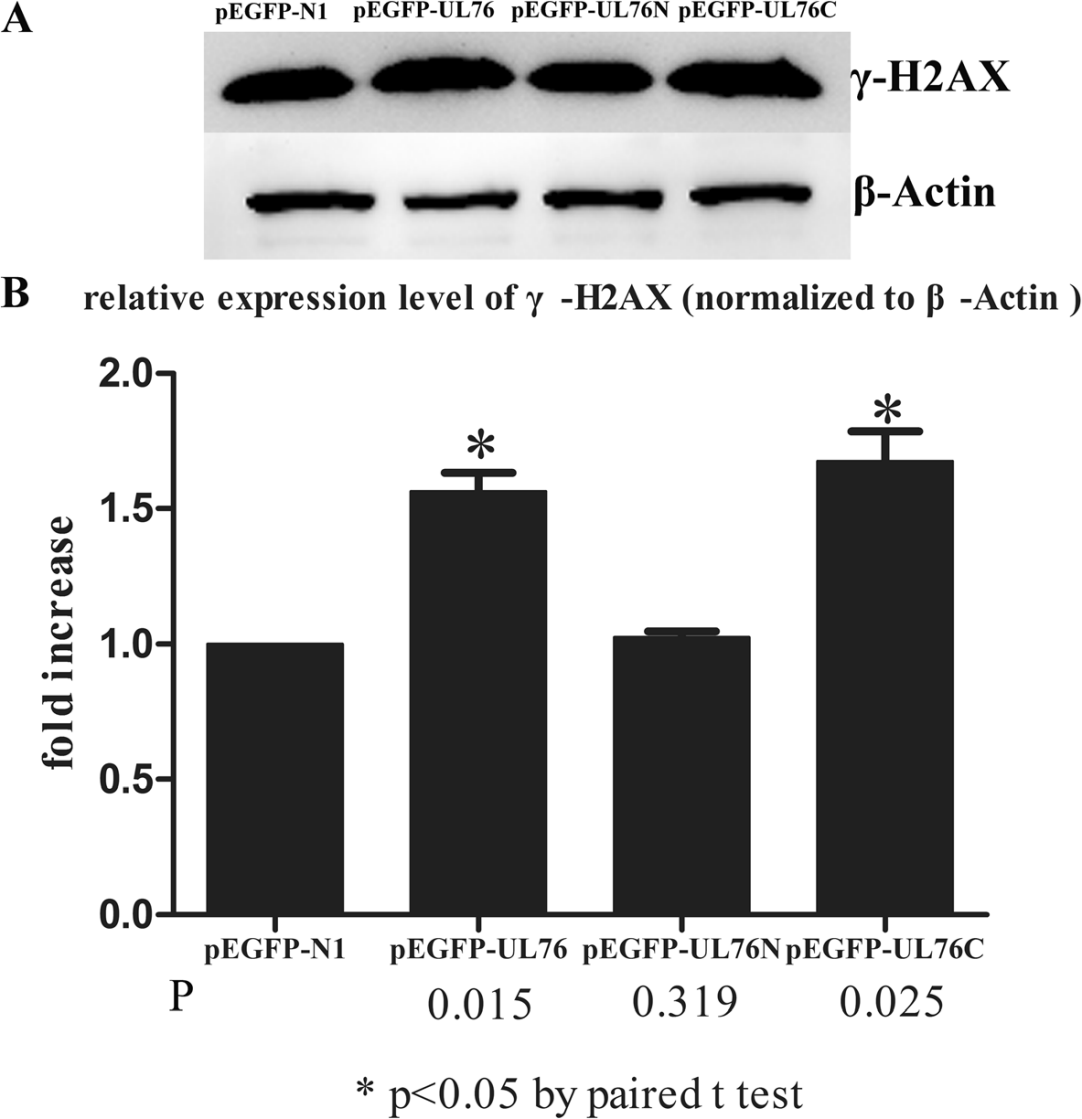
Western blot analysis of γ-H2AX protein levels in cells. β-Actin was used as a loading control. **a** Relative expression level of γ-H2AX in cells transfected with different plasmids. γ-H2AX level were increased for about 1.5 fold in cells transfected with pEGFP-UL76 or pEGFP-UL76C compared to pEGFP-N1 while pEGFP-UL76N yielded an equal level of γ-H2AX to that of pEGFP-N1. **b** Fold increase of γ-H2AX in cells transfected with pEGFP-UL76 or pEGFP-UL76C after normalization against the β-Actin loading control and the control group

Western bolting was carried out to check varied expression level of γ- H2AX in these transfected cells. The expression of γ-H2AX was increased by about 1.5 fold in HEK293 cells transfected with pEGFP-UL76 or pEGFP-UL76C compared with cells transfected with pEGFP-N1. But cells transfected with pEGFP-UL76N showed an equal level of γ-H2AX compared to the control (Fig. 4).

#### Comet assay

**Fig. 5:**
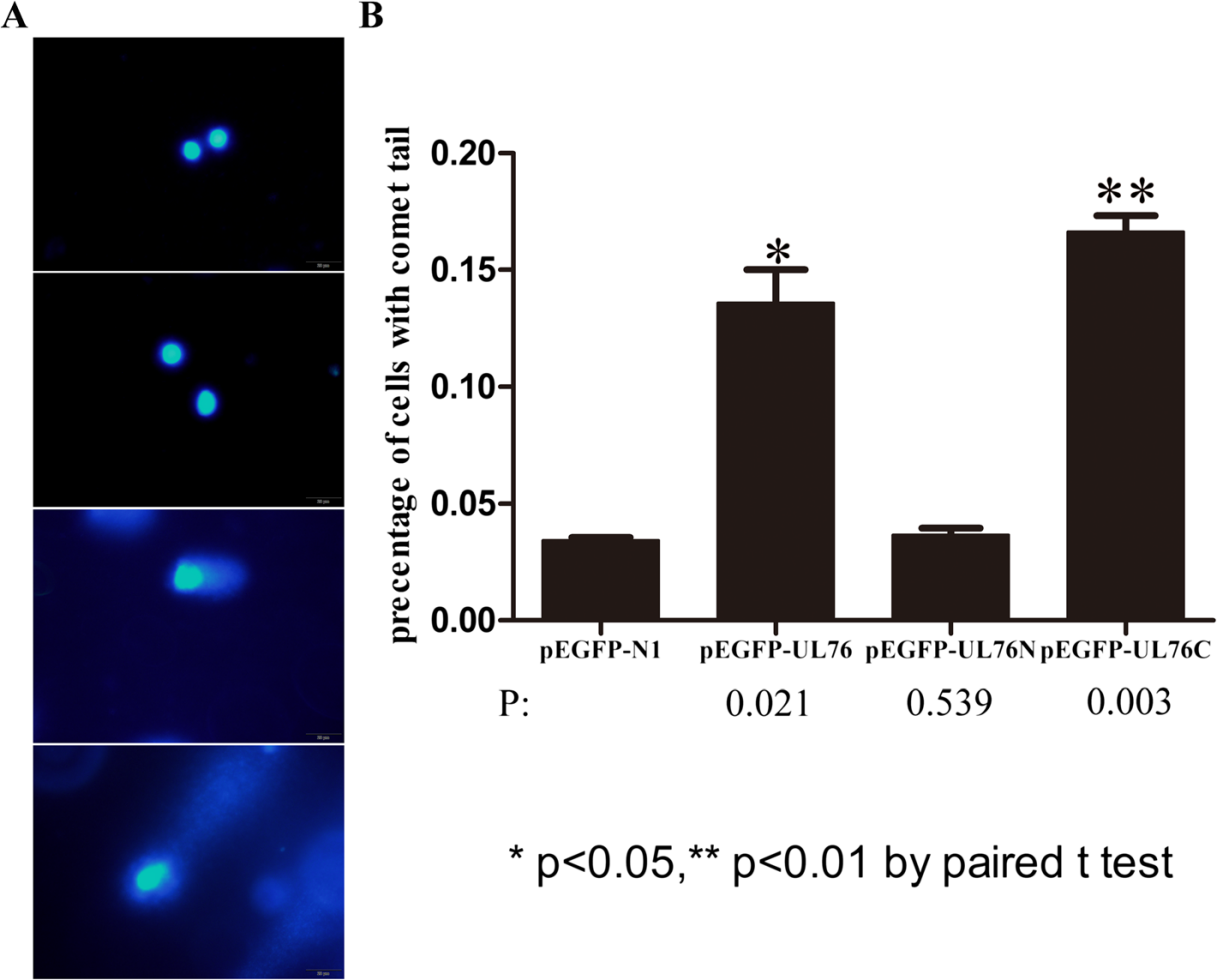
HCMV UL76 and its unconserved C terminal induced DNA breaks in *vitro*. **a** Representative images of cells without comet tails (top two panels) and with comet tails (lower two panels). **b** Ratios of cells with comet tails in each group. Each data point was calculated from three independent experiments and was plotted as a percentage of the total cell population

Comet assay was performed to investigate whether the unconserved C-terminal of UL76 was responsible for its ability to induce DNA damage. Comet assay provides a rapid and reliable way to screen genotoxic effects on a wide variety of exogenous genes in host cells. Transfected cells were lysed in an alkaline solution and were subjected to electrophoresis followed by staining with fluorescent DNA binding dye (DAPI). Cells with increased DNA damage display increased migration of chromosomal DNA from the nucleus toward the anode, which resembles the shape of a comet. For each transfection group, 200 random selective cells were examined under a fluorescence microscope at (×400) magnification. Representative images were shown in Fig. 5. Number of cells with comet tail were counted in each transfection group and average data was calculated from three independent experiments. It appears that cells with comet tails increased by 2 fold in pEGFP-UL76 transfection group and 3 fold in pEGFP-UL76C group compared to that of control. Take the efficiency of transfection into consideration, our results demonstrated that the unconserved C terminal of UL76 was the decisive region for inducing DNA damage. Although transfection efficiency is lower in pEGFP-UL76- and pEGFP-UL76C- transfected group,but indications of DNA damage were more significant than those of control group (Figs. 2, 3, 4 and 5).

## Discussion

The HCMV UL76 protein is a member of the highly conserved Herpes_UL24 family. Based on multiple protein sequence alignments of the Herpes_UL24 family, UL76, as well as other family members, was found to contain five conserved amino acid blocks at the N terminus (1-190aa) and a variable sequence at the C terminus (187-325aa). Previous studies have demonstrated that the UL76 plays many roles during virus infection including induce chromosome damage [11], elicit aggresome formation [12] and induce IL-8 expression [20]. These functions are thought to associate with virus pathogenesis. Protein integrity requires folding into appropriate three-dimensional conformation to allow the protein to perform its distinctive biological functions. Misfolded proteins will reduce motility and solubility and may produce adverse stresses in cells. Inducing DNA damage, especially double-strand DNA breaks, has a profound effect on stability of genetic information. The underlying mechanism of UL76 inducing DNA damage remains unknown. Sequence analyses indicate that the Herpes_UL24 family members contain potential endonuclease PD-(D/E)XK motifs [15]. Although the endonuclease activity has yet to be identified in any member of Herpes_UL24 family, it is believed that HCMV UL76 and UL77 proteins may be involved in the final stages of genome cleavage and packaging since they are homologs of HSV UL24 and UL25, respectively [21, 22]. We believed it would be beneficial to define which region of UL76, the conserved N terminal or the unconserved C terminal, is responsible for its ability to induce DNA damage and elicit aggresome formation. This was especially true when situations come to the functions related to cell cycle arrest. Nascimento’s team found that all representative members of Herpes_UL24 family can induce cell cycle arrest by inactivation of the cyclinB/cdc2 complex [14]. This prominent work first clarified the same role that Herpes_UL24 family members played in herpesviruses replication. This work was inspired by their previous study that found the conserved N terminal of murine gammaherpesvirus 68 ORF20, a member of conserved Herpes_UL24 family, functioned as the full-length of ORF20 [23].

In this study we obtained a different result from that of Shang-Kwei Wang’s group [12]. Our data revealed that the unconserved C terminal of UL76 was the determinant region for its ability to elicit aggresome formation. The fluorescent signal accumulates as globular aggresomes in the nuclei of cells transfected with pEGFP-UL76 or pEGFP-UL76C in our experiment. We realized that the only difference between our study and Shang-Kwei Wang’s was the expression vector used. In our experiment UL76 and its truncated fragments were constructed to pEGFP-N1 and their expression were in frame with EGFP at the N terminal. Dr. Wang’s group used pEGFP-C3 vector to express UL76 and its truncated fragments [12]. These fragments were in frame with EGFP at the C terminal. They further demonstrated that UL76 interacts with S5a, which is a major receptor of polyubiquitinated protein for UPS proteolysis via its conserved N terminal and the von Willebrand factor type A (VWA) domain of S5a by IP. Their computational analyses by AGGRESCAN and TANGO software were also in favor of their results. The predicted regions that were prone to elicit aggresome formation mainly distribute within the conserved N terminal of UL76 sequence. After carefully reviewed the publications [12], we believed we may have found where the problem came from. The 2 deletion mutations of UL76 generated by PCR were designed to generate restriction endonuclease sites *BamH*I and *EcoR*Iat 5′ end and 3′ end respectively (Material and Method in [12] and the supplement material S1 in [12]). Two deletion mutations of UL76 were also produced by PCR method. These two fragments were designed to contain restriction endonuclease sites *BamH*I and *EcoR*Iat 5′ end and 3′ end respectively. There was no problem when the mutants are expressed by pEF1/Myc-His vector. But order of the two restriction endonuclease sites, *BamH*I and *EcoR*I in the multiple cloning site (MCS) of pEGFP-C3 vector was from 3′ to 5′ direction, which will lead to the reverse of frame-reading. Therefore, it is unlikely that to these proteins were expressed as anticipated. However since we did not see the sequencing data of the constructs, we are not sure if the above explanation is correct. There might be another possibility that the two truncated fragments were mixed up at the first cloning step. It was noted that the sizes of PCR product that encodes the conserved N terminal (590 bp) and unconserved C terminal (420 bp) were similar. Testing more homologous sequences of UL76 (for example UL24 in herpes simplex virus), especially their conserved N terminal, for their ability to induce aggresome formation would be helpful to understand this controversy. Whether this controversial result was caused by different expression vectors is also need to be further studied.

## Conclusion

We have demonstrated that the unconserved C terminal of UL76 was responsible for its ability to induce DNA damage and may elicit aggresome formation.
